# Navigating climate uncertainty: employment effects of social security contributions in China

**DOI:** 10.3389/fpubh.2026.1917557

**Published:** 2026-09-25

**Authors:** Feifei Chen, Yadong Cui, Yongsheng Ji, Kaikai Wang

**Affiliations:** 1 School of Economics and Management, Shandong Youth University of Political Science, Jinan, China; 2 School of Insurance, Shandong University of Finance and Economics, Jinan, China; 3 School of Vocational Education, Shandong Youth University Of Political Science, Jinan, China

**Keywords:** climate risk, difference-in-differences, labor employment, shifting effect, social insurance contributions

## Abstract

**Introduction:**

As an institutional component of labor costs, social insurance contributions (SIC) directly shape firms’ employment decisions.

**Methods:**

Using data from China’s A-share listed companies from 2007 to 2019, and exploiting the implementation of the social insurance law (SIL) of China in 2011 as a quasi-natural experiment, this paper investigates the impact of social insurance contributions on corporate labor employment decisions and the underlying mechanisms.

**Results:**

The results show that an increase in social insurance contributions significantly reduces the scale of corporate labor employment. An analysis of the cost-shifting effect reveals that, compared with reducing wages, firms are more likely to reduce employment to alleviate the burden of social insurance contributions. Mechanism analysis indicates that higher social insurance contributions reduce labor employment through three channels: tightening liquidity constraints, reducing production scale, and increasing capital input. Heterogeneity analysis shows that the nega-tive effect of increased social insurance contributions on labor employment is more pronounced among firms with severe financing constraints and those with larger pre-existing labor employment scales.

**Discussion:**

Our findings deepen the understanding of the labor‑market consequences of social security policies and provide empirical evidence for balancing social security obligations and stable corporate employment.

## 1 Introduction

Climate change is intensifying extreme weather and degrading air quality, posing growing risks to public health and economic activity. Health risks of this kind are partly transmitted to firms through the social insurance system, under which employers are legally required to pay premiums covering medical, work injury, and pension insurance for their employees. On the one hand, this system protects workers against income and health risks; on the other hand, it constitutes a growing fixed cost for firms. Social insurance contributions are thus an institutional component of labor costs, and changes in these contributions directly affect firms’ hiring decisions. Acting as rational economic agents, firms weigh cost pressures against production needs; adjusting the scale of employment becomes an important way to alter labor costs, and this decision has a major impact on employment.

Employment is the foundation of people’s livelihoods and a key driver of development. In recent years, the Chinese government has paid close attention to employment issues. It has made clear that it wants to strengthen employment-first policies. The goal is to stabilize employment. Therefore, the research purpose of this paper is to systematically explore how institutional costs of health security systems affect corporate labor employment decisions amid growing climate uncertainty. This study helps identify a balanced path between social security provision and employment stability, and carries significant practical value and policy reference implications.

Employment is a fundamental driver of social well-being and economic development, playing a crucial role in wealth creation. Employment not only affects workers’ income and livelihoods but also serves as a critical pillar for social stability and sustainable economic development. With the growth of China’s economy, the focus of employment issues has gradually shifted from merely pursuing employment quantity to emphasizing employment quality. To enhance employment quality, China implemented the Social Insurance Law (SIL) in 2011. This marked a significant improvement in China’s social security system but also led to an increase in Social Insurance Contributions (SIC), potentially imposing heavier burdens on enterprises and influencing their labor employment decisions, thereby reducing employment levels (1).

Amid the complex and volatile domestic and global economic environments, the recovery of the global economy faces uncertainties, particularly during the post-pandemic economic reconstruction. Many countries are still struggling with weak economic growth. In China, the acceleration of population aging has caused significant changes in the labor market’s supply–demand structure, while the creation of high-quality employment opportunities continues to face considerable challenges. Although digital transformation and advancements in artificial intelligence have introduced new productivity gains for enterprises, they have also triggered a substitute effect on traditional industry jobs, leaving many low-skilled workers at risk of unemployment. Against this backdrop, employment pressures have further intensified, and the importance of employment quantity has become particularly pronounced.

China’s 2024 Central Economic Work Conference once again emphasized the importance of “ensuring employment for key groups” and called for the “implementation of detailed measures to stabilize employment”. This provides policy context for further exploration of the relationship between employment quantity and quality. Therefore, examining the impact of SIC on employment within the context of the implementation of the SIL holds significant practical and theoretical value.

The study finds that the increase in SIC brought about by SIL significantly reduces enterprises’ labor employment scale. Under the pressure of rising labor costs, enterprises are more likely to address the increased SIC burden through layoffs rather than wage reductions. The increase in SIC primarily affects enterprises’ employment decisions through mechanisms such as increased liquidity constraints, reduced production scale, and greater capital substitution. Further heterogeneity analysis reveals that the negative impact of increased SIC on enterprises’ labor employment scale is more pronounced in enterprises with severe financing constraints and larger labor employment scales.

## 2 Literature review

### 2.1 Social insurance contributions and labor employment

Enterprises’ employment decisions are influenced by numerous factors. In addition to internal factors such as production technology and management practices (2, 3), external factors like tax policies and SIC can also affect employment decisions (4–7).

The literature on the employment effects of SIC has produced divergent findings. On one side, a substantial body of research suggests that elevated SIC increase labor costs, thereby compressing firms’ employment scale. Vargas (8), for instance, documented a negative association between effective SIC rates and employee headcount in Colombian firms. Antwi and Maclean (2017) focus on health insurance. Using U.S. data, they find that increases in health insurance contributions reduce employee compensation and firms’ demand for labor, with more pronounced adverse effects on low-skilled workers (9). Similarly, Tang (10) found that higher housing provident fund contribution rates diminished firm profitability, which in turn led to reduced labor hiring. On the other side, an alternative perspective contends that social insurance programs enhance worker welfare and productivity, generating positive employment externalities (11, 12). These conflicting findings underscore the need for continued empirical scrutiny of the employment consequences of SIC, particularly within the Chinese institutional context.

Empirical evidence from China predominantly supports a negative relationship between SIC and enterprise employment. The underlying mechanisms are multifaceted. Rising SIC levels raise the effective price of labor, which may prompt firms to substitute capital for labor as a cost-minimization strategy (13, 14). Simultaneously, higher contribution burdens can tighten liquidity constraints, restricting firms’ capacity to expand operations and hire additional workers (15). Furthermore, the shifting of SIC costs may partially manifest through wage adjustments, indirectly influencing employment outcomes (16).

Despite these valuable insights, existing studies suffer from several limitations that may explain the inconsistency of findings. Theoretically, the analysis of cost-shifting mechanisms remains underdeveloped; comparative assessments of firms’ strategic choices between wage reductions and layoffs are scarce, and the behavioral motivations underlying these decisions lack systematic characterization. Empirically, the SIC rate is not strictly exogenous, introducing potential endogeneity concerns that may bias estimation results (17). For example, firms experiencing financial distress may reduce their SIC payments, generating reverse causality between contribution levels and employment outcomes. Additionally, the predominant reliance on employment growth rates as the dependent variable, while informative about changes over time, fails to capture the absolute scale of labor employment—a critical dimension for understanding the full employment effects of policy interventions. These gaps motivate the improved research design adopted in the present study.

### 2.2 Climate risk, social security, and firm employment

The escalating intensity of climate change poses both direct and indirect threats to labor productivity and public health, thereby elevating the health-related costs borne by firms (18). Extreme heat events, deteriorating air quality, and natural disasters have been shown to increase worker absenteeism, diminish on-the-job productivity, and elevate occupational health risks (19). Importantly, these climate-induced health costs are increasingly transmitted to firms through the social security system, as expenditures on medical and work injury insurance continue to rise in response to environmental stressors.

An emerging body of research at the intersection of climate risk and corporate finance indicates that firms are progressively incorporating climate-related health uncertainties into their strategic human capital decisions. Hu et al. provide evidence that heightened climate risk exposure prompts firms to adjust their human capital structures through enhanced information disclosure and strategic workforce planning, confirming a direct linkage between climate risk perception and employment adjustments. This finding resonates with the broader recognition that environmental externalities are no longer peripheral to business operations but have become central to firms’ factor input optimization (20).

Within this framework, social insurance serves as an institutional buffer against climate-induced health shocks. Desai et al. argue that integrating scientific knowledge on climate-related loss and damage with social protection systems is essential for building effective resilience mechanisms, as social protection can absorb part of the climate-induced health and economic shocks that would otherwise fall directly on firms and workers (21). However, as climate risks intensify, the increasing uncertainty regarding employee health outcomes raises firms’ expected social insurance costs. Confronted with these rising institutional expenses, rational firms optimize their factor input mix. When the marginal cost of labor exceeds its marginal benefit, reducing employment becomes a predictable adjustment channel (22). This interdisciplinary perspective provides a novel analytical lens for understanding how environmental externalities are internalized through the social security system and subsequently transmitted to labor markets, thereby bridging macro-level climate dynamics with micro-level employment decisions.

### 2.3 Literature commentary and marginal contributions

While prior studies have established a solid foundation for this research, three core gaps remain unaddressed. First, the majority of existing investigations focus on the direct employment effects of SIC in isolation, rarely incorporating climate uncertainty into the analytical framework—a limitation that impedes a comprehensive understanding of the multifaceted pressures shaping contemporary labor markets. Second, the analysis of cost-shifting mechanisms remains insufficiently developed; comparative research on the two principal adjustment paths—wage reduction versus layoff—and their respective applicability conditions is notably lacking. Third, the endogeneity of SIC indicators has not been adequately resolved in prior empirical work, and the reliance on employment growth rates as the primary outcome measure introduces measurement limitations that may obscure the full magnitude of employment effects.

Therefore, this study utilizes data from China’s A-share listed companies from 2007 to 2019 and takes the implementation of the SIL in 2011 as a quasi-natural experiment to analyze the impact of SIC on enterprises’ labor employment decisions using the Difference-in-Differences (DID) method. The potential marginal contributions of this paper are as follows: First, in terms of research mechanisms, this paper focuses on analyzing the shifting effects of SIC, particularly the behavioral logic and underlying mechanisms behind enterprises’ decisions to reduce wages or lay off workers in response to increased SIC. Unlike prior research that primarily examines the direct effects of SIC on enterprises’ costs and employment decisions, this paper emphasizes how enterprises internally adjust to external cost changes. Second, in terms of research methods, this study employs the DID model, leveraging the implementation of the SIL as an exogenous shock. This approach effectively addresses potential endogeneity issues, enhancing the reliability and robustness of the estimation results. Third, regarding research indicators, this paper uses the number of employees as a more intuitive metric that accurately reflects the absolute scale of labor employment, addressing the limitations of previous studies that overly relied on the growth rate of labor employment.

The remainder of the paper is organized as follows: Section 3 provides a mechanism analysis and presents the hypotheses to be tested; Section 4 outlines the data description and empirical design; Section 5 presents the empirical analysis of the impact of SIC on enterprises’ labor employment scale; Section 6 discusses the mechanism testing and heterogeneity analysis; and Section 7 concludes with policy recommendations.

## 3 Mechanism and hypotheses

Climate change, by intensifying extreme weather events and deteriorating environmental conditions, poses a direct and escalating threat to workers’ health and productivity. This heightened health uncertainty translates into increased operational risks for firms, manifesting as elevated absenteeism, reduced on-the-job efficiency, and higher employee turnover. To manage these mounting health-related costs and stabilize their workforce, firms rely on social insurance mechanisms—such as medical, work injury, and unemployment insurance—which serve as both a legal obligation and a strategic buffer. However, these institutional safeguards come at a cost. When climate risks drive up the health-related expenditures covered by social insurance, the financial burden on the system increases, which is consequently passed on to firms through higher statutory contribution rates or stricter enforcement. In this context, the rise in SIC represents a critical conduit through which external climate risks are internalized as a tangible labor cost pressure on enterprises.

An increase in SIC rates will raise enterprises’ labor costs, which not only compresses their profit margins and reduces their production incentives (23), thereby prompting enterprises to reduce production scale, but also increases capital investment as a substitute for labor, leading to a negative impact on labor employment. However, whether this inference holds depends on the enterprises’ ability to shift the SIC burden. Therefore, this paper first uses the theory of tax shifting to analyze how SIC affect enterprises’ employment decisions, concluding that enterprises face significant difficulty in shifting the SIC burden. It then elaborates on the mechanisms through which SIC affect enterprises’ labor employment decisions, including liquidity constraints, production scale, and capital substitution, and proposes hypotheses to be tested in the subsequent empirical analysis.

### 3.1 Shifting effect: a comparison of wage reduction and layoffs

Faced with the increase in SIC, enterprises may adjust their factor inputs, which in turn can affect the number of workers they hire. In general, an enterprise’s labor costs are determined by the number of employees and their compensation. To alleviate the burden of SIC, enterprises may consider reducing either the number of employees or the wages of their workers. However, according to the theory of tax shifting, the relative ease of implementing these adjustments depends on the comparison between the elasticity of labor demand and the elasticity of labor supply. If the demand elasticity is smaller than the supply elasticity, enterprises can more easily shift the burden of SIC onto employees by reducing wages, without significantly altering the number of workers they employ. In this case, enterprises would prefer wage reductions as a method of cost management. On the other hand, if the elasticity of labor demand exceeds the elasticity of labor supply, enterprises would find it more difficult to reduce wages without affecting the number of employees. In this scenario, layoffs would become the primary mechanism for adjusting to the increased SIC burden.

Figure 1 illustrates the allocation of labor employment quantity and employee wages when enterprises face different labor demand elasticities, assuming labor supply elasticity remains unchanged. When the enterprise faces a demand curve D_1_, labor demand elasticity is lower than labor supply elasticity. In contrast, when enterprise faces a demand curve D_2_, labor demand elasticity exceeds labor supply elasticity. Initially, the employee wage and labor employment numbers are set at W and E, respectively. When enterprises are required to pay SIC for each employee, the actual wage paid to employees will exceed W, causing the demand curve to shift leftward. Thus, the demand curves D_1_ and D_2_ shift to D_11_ and D_21_, respectively. From a comparative static perspective, enterprises can choose to reduce labor employment or wages to alleviate the burden of SIC.

**Figure 1:**
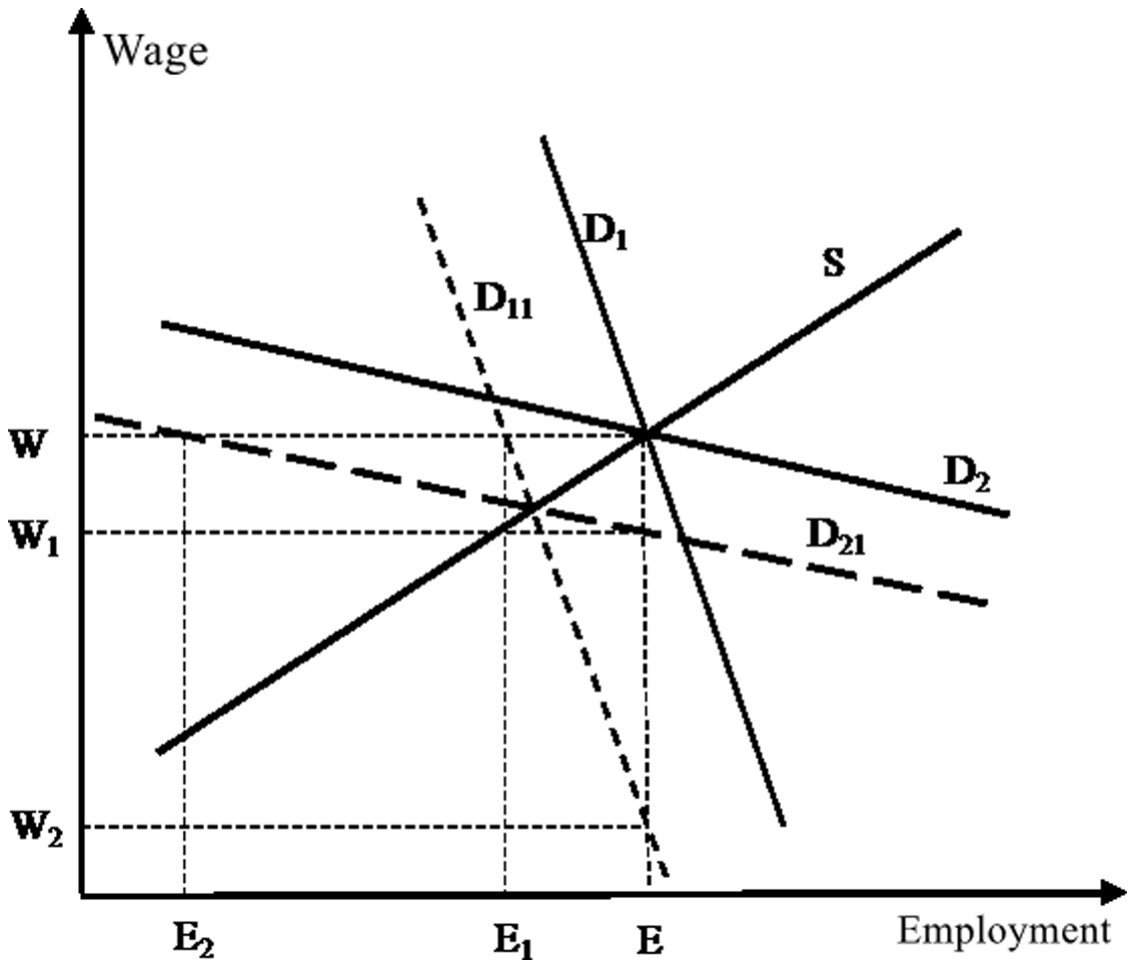
Impact of SIC on enterprises’ labor employment decisions.

When enterprises aim to maintain the same level of labor employment while reducing wages to offset the increased SIC, the shift in the labor demand curve depends on the relative elasticities. When the demand elasticity is smaller than labor supply elasticity, an increase in SIC shifts the demand curve from D_1_ to D_11_, requiring enterprises to reduce employee wages from W to W_2_. On the other hand, when labor demand exceeds labor supply elasticity, an increase in SIC shifts the demand curve from D_2_ to D_21_, and enterprises are required to reduce wages from W to W_1_. When labor demand elasticity is greater than labor supply elasticity, reducing wages becomes more difficult, and enterprises are more likely to resort to layoffs as a means of alleviating the burden of SIC (Figure 2).

**Figure 2:**
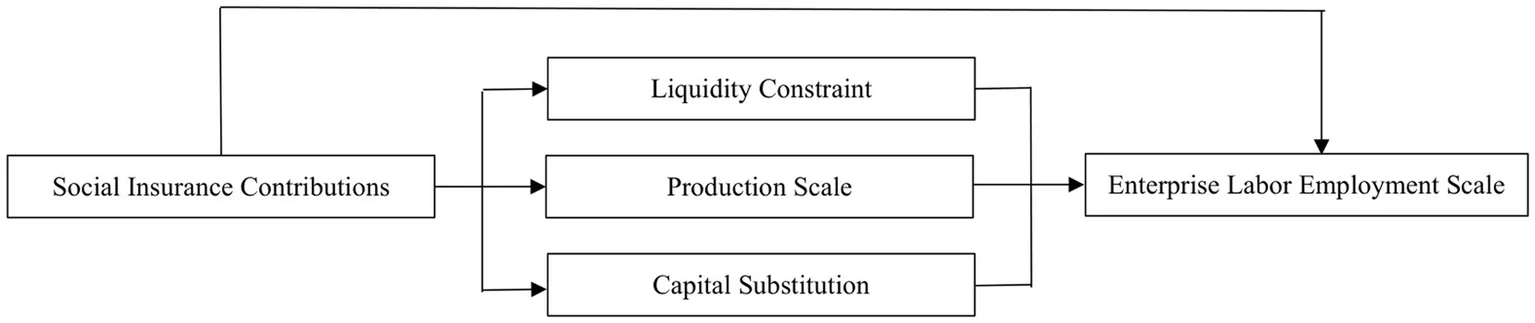
Mechanism of SIC affecting enterprise labor employment scale.

Similarly, if enterprises do not adjust employee wages and instead attempt to alleviate the SIC burden through layoffs, the effect depends on the relative elasticities. When the demand elasticity is lower, an increase in SIC shifts the labor demand curve from D_1_ to D_11_, and enterprises would need to reduce the number of employees from E to E_1_. However, when the labor demand is higher, an increase in SIC shifts the labor demand curve from D_2_ to D_21_. If employee wages remain unchanged, the increased SIC will reduce the number of employees from E to E_2_. It can be observed that when labor demand elasticity is greater than labor supply elasticity, enterprises tend to lay off a larger proportion of employees in response to rising SIC.

In summary, whether enterprises choose layoffs or wage reductions to cope with increased SIC depends on the comparison of labor demand and supply elasticities in the labor market. Based on this, this paper proposes the following hypotheses:

*H1a*: When labor demand elasticity is greater than labor supply elasticity, enterprises are more likely to choose layoffs in response to rising SIC levels.

*H1b*: When labor demand elasticity is smaller than labor supply elasticity, enterprises are more likely to choose wage reductions in response to rising SIC levels.

### 3.2 Mechanism of SIC affecting enterprises’ labor employment scale

#### 3.2.1 Liquidity constraint

In essence, an increase in SIC rates can be viewed as an additional tax burden imposed on enterprises, which reduces their available capital and exacerbates their liquidity constraints (5). Prior to the SIL, regulatory oversight over SIC was weak, and the enforcement of contribution standards was inconsistent, leading to widespread noncompliance by enterprises, such as tax evasion, underreporting, and underpayment. This noncompliance resulted in actual contribution rates being significantly lower than the statutory rates, allowing enterprises to save substantial amounts of available capital, thereby alleviating their liquidity constraints. In this context, enterprises were able to utilize the saved funds to acquire more production factors, thereby expanding their production capacity.

However, after the implementation of the SIL, with the SIC enforcement system becoming more standardized and stringent, enterprises’ noncompliance with SIC faced severe penalties. The actual contribution rates of enterprises will tend to align with the statutory rates. In this scenario, enterprises must allocate additional funds from their available capital to pay for SIC, which reduces their remaining cash flow after fulfilling essential production expenses, leading to liquidity constraints (24). Furthermore, the increase in SIC can lead to higher operational leverage and weaken an enterprise’s ability to service its debt, thereby lowering banks’ expectations regarding the enterprise’s future profitability. As a result, banks become more cautious in extending loans, increasing the difficulty for enterprises to obtain credit, which further exacerbates their liquidity constraints (25). Thus, the second hypothesis is:

*H2*: An increase in SIC rates will intensify liquidity constraints for enterprises, potentially leading to a reduction in labor employment levels.

#### 3.2.2 Production scale

The previous analysis indicates that after the SIL, due to the limited ability of enterprises to shift SIC, additional social insurance costs are needed for employees on top of their original wages. Moreover, under the constraints of minimum wage regulations and labor contract requirements, the increase in SIC represents a continuous fixed cost for enterprises (22). This increases the pressure on enterprises, reducing their production scale and labor employment levels. Specifically, on the one hand, the rise in labor costs due to higher SIC will inevitably squeeze profit margins and lead to operational cash flow difficulties (26), restricting investments in areas such as product research and development, production equipment upgrades, and market expansion. This will cause enterprises to reduce production scale (27). In this scenario, enterprises are likely to reduce new employee recruitment or even resort to layoffs to alleviate the burden of SIC, ultimately reducing labor employment levels (10). On the other hand, in competitive product markets, enterprises facing higher SIC may choose to raise product prices to maintain unit product profits. However, price increases will reduce market demand for the product, potentially leading to unsold inventory. In such cases, enterprises will have to scale back production and product output to match market demand, which will also lead to a reduction in labor employment levels (28). Therefore:

*H3*: An increase in SIC rates will lead enterprises to reduce their production scale, thereby resulting in a negative effect on labor employment levels.

#### 3.2.3 Capital substitution

According to the factor substitution theory, when the labor price increases relative to capital, enterprises, driven by the goal of profit maximization, will increase their capital input to substitute the relatively expensive labor factor (29). As an important component of labor costs, the implementation of SIL directly increases the social insurance payment burden on enterprises, which in turn raises the labor cost. Since enterprises cannot fully smooth this labor cost increase through wage reductions, they will opt to increase capital inputs, such as purchasing more advanced machinery and introducing automated production lines to replace some of the labor employment (13). Compared to labor, the marginal cost of using machinery and equipment in the subsequent stages is very low, and this cost does not increase with the rise in labor costs. Machinery only needs to complete standardized production tasks according to preset procedures. The heavier the social insurance payment burden on enterprises, the more they can reduce labor costs and achieve higher marginal returns by increasing capital inputs (14). Additionally, the increase in social insurance payments will also encourage enterprises to focus on advanced technologies such as artificial intelligence, promoting technological innovation, upgrading production processes, and improving product quality through increased research and development investments. This not only enhances the enterprise’s profit level and core competitiveness but also reduces its dependence on labor employment, achieving both cost reduction and efficiency improvement (30). Therefore:

*H4*: An increase in SIC rates will encourage enterprises to adopt more capital to substitute labor, thereby reducing the number of labor hires.

To facilitate understanding, a framework diagram for the mechanism is shown below:

## 4 Methods

### 4.1 Model

This study adopts a quantitative, quasi-experimental research design. The SIL was mandated by national legislation, took effect at a single point in time (July 2011), and applied uniformly across regions and industries; its implementation is therefore plausibly exogenous to any individual firm’s employment decisions and can serve as a quasi-natural experiment. Drawing on the difference-in-differences (DID) approach employed in the literature on policy shocks to labor costs e.g., (31–33), the empirical analysis proceeds in four steps. First, firms are classified into treatment and control groups according to their pre-policy labor intensity, and a multi-way fixed-effects DID model is estimated with standard errors clustered at the firm level. Second, the parallel-trend assumption underlying the DID identification is examined using an event-study specification. Third, a battery of robustness checks is conducted, including PSM-DID estimation, a placebo test based on random treatment assignment, alternative grouping criteria, and the exclusion of confounding policy shocks. Fourth, mechanism and heterogeneity analyses are performed to illuminate the channels through which SIC affect enterprise labor employment. The data are derived from the financial statements of China’s A-share listed companies, and the detailed variable definitions and sample construction are described in the remainder of this section. This study treats the implementation of 2011 SIL as an exogenous policy shock and empirically analyzes how SIC affect enterprise labor employment with a DID model. The econometric model is specified as follows:

$$\text{Labo}r_{\text{ijpt}}=\alpha_{0}+\text{βTrea}t_{i}*\mathrm{Pos}t_{t}+\gamma X_{\text{ijpt}}+\mu_{i}+\vartheta_{j}+\epsilon_{p}+\delta_{t}+\varepsilon_{\text{ijpt}}$$ (1)

The subscripts *i*, *j*, *p*, and *t* are the enterprise, industry, province, and year, respectively. *Labor_ijpt_* is enterprise’s labor employment. *Post_t_* is 1 if the enterprise’s sample is in or before 2011, and 0 otherwise. *Treat_i_* is 1 for treatment group and 0 for control group. Treat_i_*Post_t_ is the policy implementation variable, which is the key focus of this study. β represents the estimated coefficient of the policy implementation variable, and the expected sign is negative. *X_ijpt_* denotes other factors influencing enterprise labor employment. $\mu_{i}$ , $\vartheta_{j}$ , $\epsilon_{p}$ , and $\delta_{t}$ represent enterprise, industry, province, and year fixed effects, respectively.

### 4.2 Variables

#### 4.2.1 Dependent variable

The enterprise’s labor employment (*Labor*) is the number of employees from the financial statements of listed enterprises. To reduce the volatility of the data, the natural logarithmic form is used in this study.

#### 4.2.2 Treatment group and control group

Given that the SIL occurred at a single time point and uniformly across regions, it is not possible to naturally derive a treatment group and a control group from real-world data. Since SIC are a key component of enterprise labor costs, the impact of SIL is likely to be more significant for labor-intensive enterprises. Therefore, drawing on the approaches of Kim, Campello and Larrain, and Serfling, this paper groups enterprises over their labor intensity in 2010 (before the law’s implementation) (31–33).

The ratio of employee compensation to sales revenue is used to define labor intensity. The specific method for dividing the treatment and control groups is as follows: the sample of enterprises in 2010 is ranked in ascending order of labor intensity and then split into three equal parts. Enterprises in the lowest third are in the control group, with 0 for *Treat_i_*, while those in the highest third in the treatment group, with a value of 1 for *Treat_i_*. Firms in the middle third of the labor intensity distribution are excluded from the baseline sample to ensure a clean treatment–control distinction. This approach follows the extreme group design common in the DID literature. As robustness checks, Tables 1, 2 confirm that the results are not sensitive to this sample restriction.

**Table 1:** PSM-DID estimation results.

	Kernel matching		Radius calipers matching		Nearest neighbor matching	
	(1)	(2)	(3)	(4)	(5)	(6)
Treat*Post	−0.1293^***^	−0.1290^***^	−0.1659^***^	−0.1655^***^	−0.1386^***^	−0.1383^***^
	(0.0421)	(0.0423)	(0.0429)	(0.0431)	(0.0421)	(0.0422)
Control variables	Yes	Yes	Yes	Yes	Yes	Yes
Province/industry FE		Yes		Yes		Yes
Enterprise/year FE	Yes	Yes	Yes	Yes	Yes	Yes
*N*	13,899	13,899	11,108	11,108	13,660	13,660
Adjusted-*R^2^*	0.8977	0.8969	0.8919	0.8909	0.8943	0.8935
F-statistic	99.6170	98.9262	96.0385	95.1801	100.0365	99.3192

For ease of analysis, control variables are not listed here. *, **, and *** represent significance at the 10, 5, and 1% levels, respectively. The enterprise-level cluster robust standard errors are in parentheses.

**Table 2:** Estimation with alternative grouping method.

	Firm & Year FE (Baseline)	+Industry FE	+Province FE	+Industry & Province FE
	(1)	(2)	(3)	(4)
Treat*Post	−0.0908^**^	−0.0908^**^	−0.0908^**^	−0.0908^**^
	(0.0370)	(0.0371)	(0.0371)	(0.0371)
Industry FE		Yes		Yes
Province FE			Yes	Yes
Control variables	Yes	Yes	Yes	Yes
Enterprise FE	Yes	Yes	Yes	Yes
Year FE	Yes	Yes	Yes	Yes
*N*	14,002	14,002	14,002	14,002
Adjusted-*R*^2^	0.9001	0.8996	0.8999	0.8994
F-statistic	117.0831	116.5053	116.8319	116.2540

For ease of analysis, control variables are not listed here. *, **, and *** represent significance at the 10, 5, and 1% levels, respectively. The enterprise-level cluster robust standard errors are in parentheses.

#### 4.2.3 Control variables

Previous studies have suggested that factors such as enterprise size, debt structure, profitability, and ownership structure may all influence employment decisions (15). Therefore, following existing research, the following variables are control variables: enterprise size (*Size*), represented by the natural logarithm of total assets; debt ratio (*Debt*), represented by the ratio of total liabilities to total assets; profitability (*ROA*), represented by the return on assets; enterprise age (*Eage*), calculated as the survey year minus the year of establishment; ownership nature (*Ownership*), represented by whether the enterprise is state-owned; enterprise growth (*Growth*), is the annual growth rate of sales revenue; and ownership concentration (*Concentration*), is the shareholding ratio of the largest shareholder.

After data processing, the study obtained a total of 13,904 enterprise samples (6,348 in control and 7,556 in treatment). The data used in this study is sourced from the A-share data of Chinese listed companies. Considering the accounting standard changes in 2006, to avoid dimensional inconsistency, the sample period selected is from 2007 to 2019, which conveniently includes the implementation of the 2011 SIL. The following data processing steps were conducted: (1) Financial, real estate, ST, and *ST listed companies were excluded; (2) Companies with serious data missing were removed; (3) Companies listed after 2011 were excluded; (4) All continuous variables were winsorized at the 1% level at both ends and their natural logarithms were taken to avoid the potential influence of outliers. Table 3 reports the data characteristics of the key variables.

**Table 3:** Descriptive statistics.

Variable	Mean	Standard deviation	Minimum	Maximum
Labor	7.7897	1.3208	4.1431	11.2861
Size	22.1962	1.3252	19.5268	26.2151
Debt	0.4401	0.2030	0.0484	0.8831
ROA	0.0483	0.0404	0.0013	0.2106
Eage	2.6888	0.4103	1.3863	3.4012
Ownership	0.4658	0.4988	0	1
Growth	0.2343	0.5640	−0.4887	4.1724
Concentration	3.4612	0.4704	2.1849	4.3159

## 5 Empirical results

### 5.1 Baseline estimation result

Table 2 reports the regression results estimated from Equation (1). In Table 4, the coefficient of Treat*Post represents the estimated effect of the increase in SIC levels on enterprise labor employment. To ensure the robustness of the estimation results, this study employs a stepwise regression approach, adding different control variables to the regression sequentially. The final estimation results are reported in Column (7). The coefficient of Treat*Post is negative and significant. So increased SIC levels resulting from the SIL has led to a reduction in enterprise labor employment. Economically, the results in Column (7) suggest that after the SIL, the increase in SIC has led to a 11.92% decrease in enterprise labor employment. Hypothesis 1 is partially validated. Among the control variables, the effects of enterprise size, debt ratio, and ownership nature are significantly positive, indicating that state-owned enterprises or larger enterprises typically have more production capacity, broader market demand, and greater financial stability, allowing them to provide more employment opportunities. Meanwhile, highly leveraged enterprises may expand through borrowing, increasing production and operational activities, which require more labor to support their operations. One potential concern is whether the control variables constitute “bad controls”—that is, whether they are themselves affected by the SIL and thus absorb part of the policy’s causal effect. Three considerations alleviate this concern. First, as shown by the stepwise design in Table 4, the Treat*Post coefficient is remarkably stable across Columns (1)–(7), ranging from −0.1238 to −0.1310; if the controls were absorbing the treatment effect, the coefficient would change substantially as controls are added. Second, most of the controls—firm size, firm age, ownership type, and ownership concentration—are predetermined or adjust slowly over time, making them unlikely to respond to the SIL within the same year. Third, this control structure is standard in the literature on social insurance contributions and employment e.g., Kugler and Kugler (17) and Benzarti and Harju (21).

**Table 4:** Impact of SIC on enterprise labor employment scale.

	Progressively adding control variables	(1)	(2)	(3)
(4)	(5)	(6)	(7)
Treat*Post	−0.1238^***^	−0.1282^***^	−0.1298^***^	−0.1310^***^	−0.1280^***^	−0.1274^***^	−0.1269^***^
	(0.0429)	(0.0425)	(0.0427)	(0.0426)	(0.0426)	(0.0426)	(0.0426)
Size	0.6840^***^	0.6640^***^	0.6638^***^	0.6637^***^	0.6610^***^	0.6615^***^	0.6607^***^
	(0.0264)	(0.0275)	(0.0275)	(0.0275)	(0.0275)	(0.0277)	(0.0275)
Debt		0.3130^***^	0.2995^***^	0.2945^***^	0.2886^***^	0.2899^***^	0.2929^***^
		(0.0932)	(0.0948)	(0.0964)	(0.0952)	(0.0954)	(0.0950)
ROA			−0.2227	−0.2224	−0.1937	−0.1841	−0.1840
			(0.2599)	(0.2599)	(0.2612)	(0.2653)	(0.2653)
Eage				0.0377	0.0371	0.0366	0.0494
				(0.1026)	(0.1021)	(0.1022)	(0.1051)
Ownership					0.1501^*^	0.1498^*^	0.1519^*^
					(0.0869)	(0.0868)	(0.0864)
Growth						−0.0030	−0.0036
						(0.0129)	(0.0130)
Concentration							0.0366
							(0.0524)
Firm FE	Yes	Yes	Yes	Yes	Yes	Yes	Yes
Year FE	Yes	Yes	Yes	Yes	Yes	Yes	Yes
Province FE	Yes	Yes	Yes	Yes	Yes	Yes	Yes
Industry FE	Yes	Yes	Yes	Yes	Yes	Yes	Yes
*N*	13,904	13,904	13,904	13,904	13,904	13,904	13,904
Adjusted-*R*^2^	0.8958	0.8964	0.8964	0.8964	0.8966	0.8966	0.8966
F-statistic	336.6903	237.5887	181.7418	150.0420	127.3498	109.3894	97.7177

*, **, and *** represent significance at the 10, 5, and 1% levels, respectively. The enterprise-level cluster robust standard errors are in parentheses.

### 5.2 Robustness test

#### 5.2.1 Parallel trend test

Theoretically, the parallel trend assumption is a prerequisite for DID method. This study employs an event study approach for the parallel trend test. The econometric model is specified as follows:

$$\begin{array}{l} \text{Labo}r_{\text{ijpt}}=\alpha_{0}+\beta \sum_{t=2007,t\neq 2011}^{2019}\text{Trea}t_{i}*T_{t}+\gamma X_{\text{ijpt}}\\ +\mu_{i}+\vartheta_{j}+\epsilon_{p}+\delta_{t}+\varepsilon_{\text{ijpt}} \end{array}$$ (2)

where *T_t_* is the policy shock variable, which is 1 for year *t* and 0 for other years. Figure 3 is generated from the estimation results of Equation (2). The estimated coefficients of Treat_i_*T_t_ are not significant prior to 2011, while after 2011, the estimated coefficients are negative and highly significant. This indicates that before the SIL, there were no significant differences in the changes in enterprise labor employment between the two groups. Therefore, this study constructs a DID model to empirically test the impact of SIC, and the results are consistent with the parallel trend assumption, making the methodology reasonable. We note that 2011 serves as the reference period in the event study. Since the SIL was implemented in July 2011, this is a partially treated year. Readers concerned about this issue are referred to Table 5, Column (1), which excludes all 2011 observations entirely and yields consistent results (−0.1407, *p* < 0.01).

**Figure 3:**
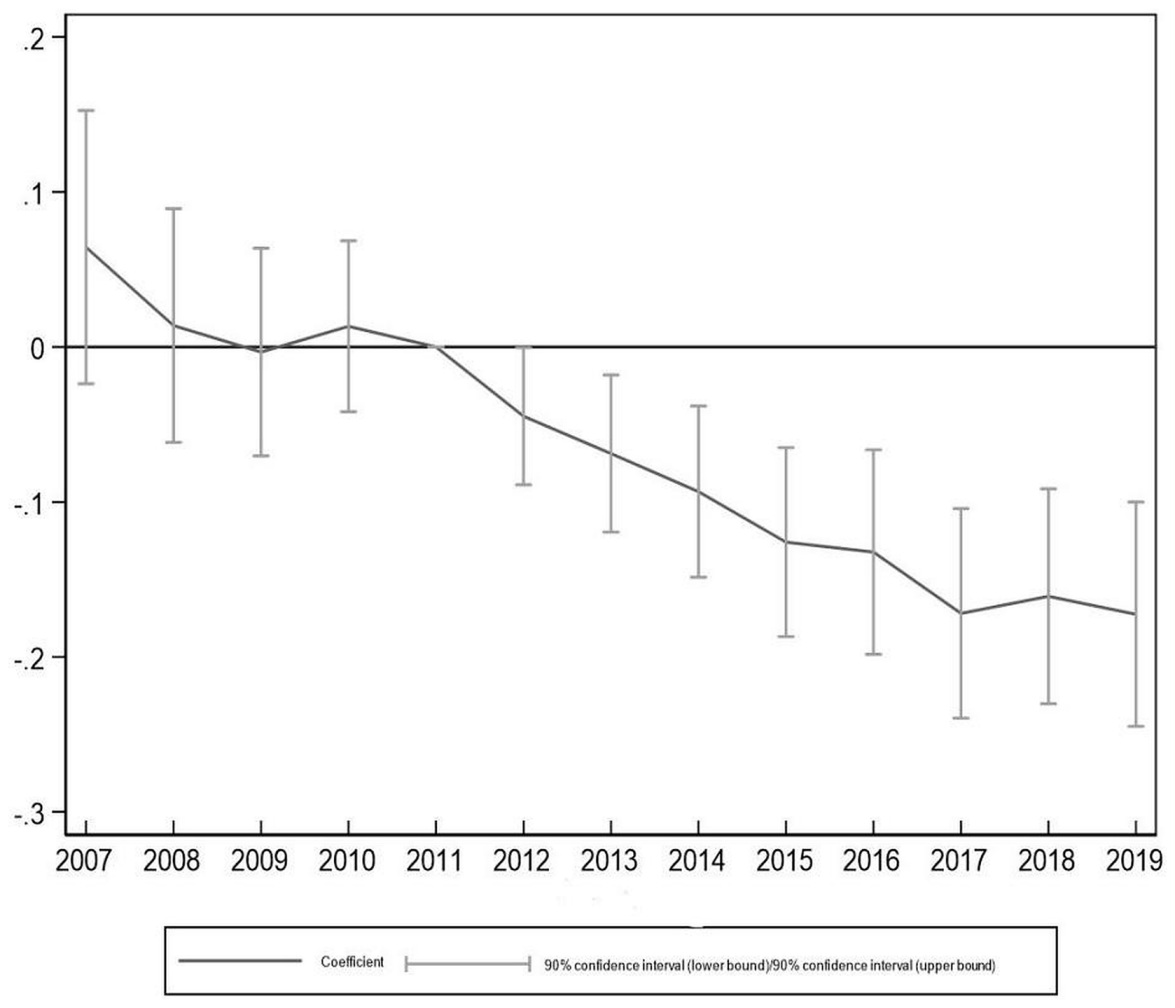
Parallel trend test. The reference period is 2011. Results are robust to excluding 2011 entirely (see Table 5, Column 1).

**Table 5:** Estimation after excluding other potential factors.

	Re-estimation Excluding 2011 Firm Observations	Industry × Year Fixed Effects	Province × Year Fixed Effects
	(1)	(2)	(3)
Treat*Post	−0.1407^***^	−0.2023^***^	−0.1145^***^
	(0.0455)	(0.0468)	(0.0440)
Control variables	Yes	Yes	Yes
Industry*Year FE	–	Yes	–
Province*Year FE	–	–	Yes
Province FE	Yes	–	Yes
Industry FE	Yes	Yes	–
Firm FE	Yes	Yes	Yes
Year FE	Yes	Yes	Yes
*N*	12,833	13,825	13,904
Adjusted-*R*^2^	0.8962	0.9003	0.8976
F-statistic	97.5907	99.1580	100.4322

*, **, and *** represent significance at the 10, 5, and 1% levels, respectively. The enterprise-level cluster robust standard errors are in parentheses.

#### 5.2.2 Alternative research methods

Generally, enterprises with higher labor intensity tend to allocate a larger proportion of their costs to labor, so when facing an increase in SIC burdens, these enterprises may experience more significant changes in labor employment levels. Therefore, dividing the treatment and control groups based on labor intensity is no longer random and could lead to endogeneity issues arising from sample selection bias. Currently, the propensity score matching-difference in differences (PSM-DID) method is an effective solution to address the issue of selection bias endogeneity. Thus, this paper further uses the PSM-DID method to test the impact of SIC.

First, the treatment variable is used as the dependent variable, and the enterprise characteristics, including the control variables used in this study, are used as independent variables to perform a Logit regression to estimate the propensity score for each enterprise to be treated. Second, the PSM is applied to match the treatment and control group samples so that they are as similar as possible before the implementation of the SIL, thereby correcting the selection bias endogeneity issue. Finally, the DID method is used to re-examine the impact of SIC. The estimation results with three matching methods are shown in Table 1. Compared to the baseline estimation results, the estimated parameters obtained using the PSM-DID method remain significantly negative at the 1% significance level, further confirming the robustness of the baseline estimation results.

#### 5.2.3 Excluding possible exogenous shocks

##### 5.2.3.1 Excluding pre-treatment factors

Before the implementation of the SIL, other events in 2008 and 2009 may also have influenced the enterprise’s labor employment decisions. First, the Labor Contract Law in 2008 strengthened the protection of workers’ rights. Second, the global financial crisis significantly impacted the proportion of production factor inputs in enterprises. To exclude the effects of these two events, a dummy variable was constructed, where samples from 2008 and subsequent years are 1, and samples from previous years are 0. This dummy variable was then interacted with the treatment variable and included in Equation 4 for re-estimation, with the results shown in Column (1) of Table 6. Third, in 2009, China switched from a production-based value-added tax to a consumption-based value-added tax, which may have also affected enterprise input decisions, thus altering labor employment decisions. Similarly, a dummy variable was constructed where samples in 2009 or later are 1, and previous years are 0. This dummy variable also interacted with the treatment variable and was included in the Equation 4 for re-estimation, with the results presented in Column (2) of Table 6. As shown, after accounting for the implementation of the Labor Contract Law, the financial crisis, and the value-added tax reform, Treat*Post’s estimated coefficient remained significantly negative, confirming that the increase in SIC still significantly reduced enterprise labor employment scale.

**Table 6:** Estimation after excluding possible exogenous shocks.

	Labor Contract Law / Financial Crisis	VAT Reform	Excluding Years after 2017	Excluding Years after 2015	Excluding Years after 2014
	(1)	(2)	(3)	(4)	(5)
Treat*Post	−0.1138^***^	−0.1112^***^	−0.1131^***^	−0.0885^**^	−0.0713^*^
	(0.0410)	(0.0406)	(0.0402)	(0.0383)	(0.0375)
Labor contract law/financial crisis	Yes				
Value-added tax reform		Yes			
Control variables	Yes	Yes	Yes	Yes	Yes
Province FE	Yes	Yes	Yes	Yes	Yes
Industry FE	Yes	Yes	Yes	Yes	Yes
Enterprise FE	Yes	Yes	Yes	Yes	Yes
Year FE	Yes	Yes	Yes	Yes	Yes
*N*	13,904	13,904	11,535	9,031	7,863
Adjusted-*R*^2^	0.8966	0.8966	0.9007	0.9122	0.9185
F-statistic	86.8302	86.8195	78.6648	46.7530	32.2467

*, **, and *** represent significance at the 10, 5, and 1% levels, respectively. The enterprise-level cluster robust standard errors are in parentheses.

##### 5.2.3.2 Excluding post-treatment factors

After the implementation of the SIL, several other events emerged that could have impacted enterprises’ labor employment decisions. First, in 2018, China adjusted its social insurance contribution collection mechanism, transferring the duty for collection, previously shared between the tax bureau and social insurance agencies, to a centralized system managed solely by the tax authorities. This institutional change strengthened the enforcement of contribution collections and curtailed enterprises’ ability to underreport or evade payments. To control this policy interference, this paper excludes enterprise samples from 2017 onwards and re-estimates the model. The estimation results are presented in Column (3) of Table 6. Second, in 2016, China implemented a policy to temporarily reduce SIC rates, alleviating the social insurance payment burden on enterprises. Thus, this study excludes enterprise samples from 2015 onwards and re-estimates the model. The estimation results are shown in Column (4) of Table 6.

Third, the Chinese government began implementing supply-side structural reforms in 2015. To account for the potential effects of this reform, this study excludes enterprise samples from 2014 onwards and re-estimates the model. The estimation results are presented in Column (5) of Table 6. As seen from the results, even after accounting for the above post-implementation factors, the interaction term Treat*Post remains significantly negative, indicating that the increase in SIC still significantly reduced the labor employment scale of enterprises.

##### 5.2.3.3 Excluding other potential factors

In addition to the aforementioned event shocks, other potential factors may influence enterprise labor employment decisions. First, since the implementation of the SIL occurred in July, and this paper defines the policy implementation year on an annual basis, samples from the implementation year may include confounding factors. Therefore, this paper removes the enterprise samples in 2011 and re-estimates the model in Column (1) of Table 5. Second, the time-varying characteristics of the province and industry in which the enterprise is located may also affect its labor employment scale. Thus, this paper incorporates the interaction fixed effects of industry and year, as well as province and year, into Equation 4. The estimated results are in Columns (2) and (3) of Table 5, respectively. The estimation results in Table 5 indicate that, after considering the aforementioned potential influencing factors, the estimated coefficient of the interaction term remains negative and significant. So the increase in social insurance payment levels caused by the SIL still leads to a reduction in enterprise labor employment scale, further validating Hypothesis 1.

##### 5.2.3.4 Alternative grouping method

In the baseline estimation, this paper primarily uses the labor intensity of enterprises in 2010 to divide the treatment and control groups. However, if the data from that year contains outliers, this method of classification may no longer be appropriate. To mitigate the potential issues caused by large fluctuations in the data for specific years, this study instead uses the average labor intensity of enterprises from 2007 to 2010 (before the SIL), as the basis for classification. Specifically, if the average labor intensity of enterprise i between 2007 and 2010 falls within the upper third of the distribution, Treati is assigned a value of 1; if it falls within the lower third, Treati is assigned a value of 0. Based on these redefined treatment and control groups, this paper reexamines the impact of SIL on enterprise labor employment. As shown in Table 2, while the significance of Treat*Post decreases somewhat, the fundamental conclusion, that the increased levels reduces the scale of enterprise labor employment, remains unchanged.

#### 5.2.4 Placebo test

This paper also conducts a counterfactual analysis through random sampling for further validation. The distribution of estimated coefficients obtained from 500 repeated random samplings is shown in Figure 4. It can be observed that most of the interaction term estimates are concentrated around zero, with significance levels generally lower than 10%. At the same time, the baseline estimated coefficient of −0.1269 falls outside of this range. These results suggest that the estimation results are unlikely to be disturbed by other unobserved factors.

**Figure 4:**
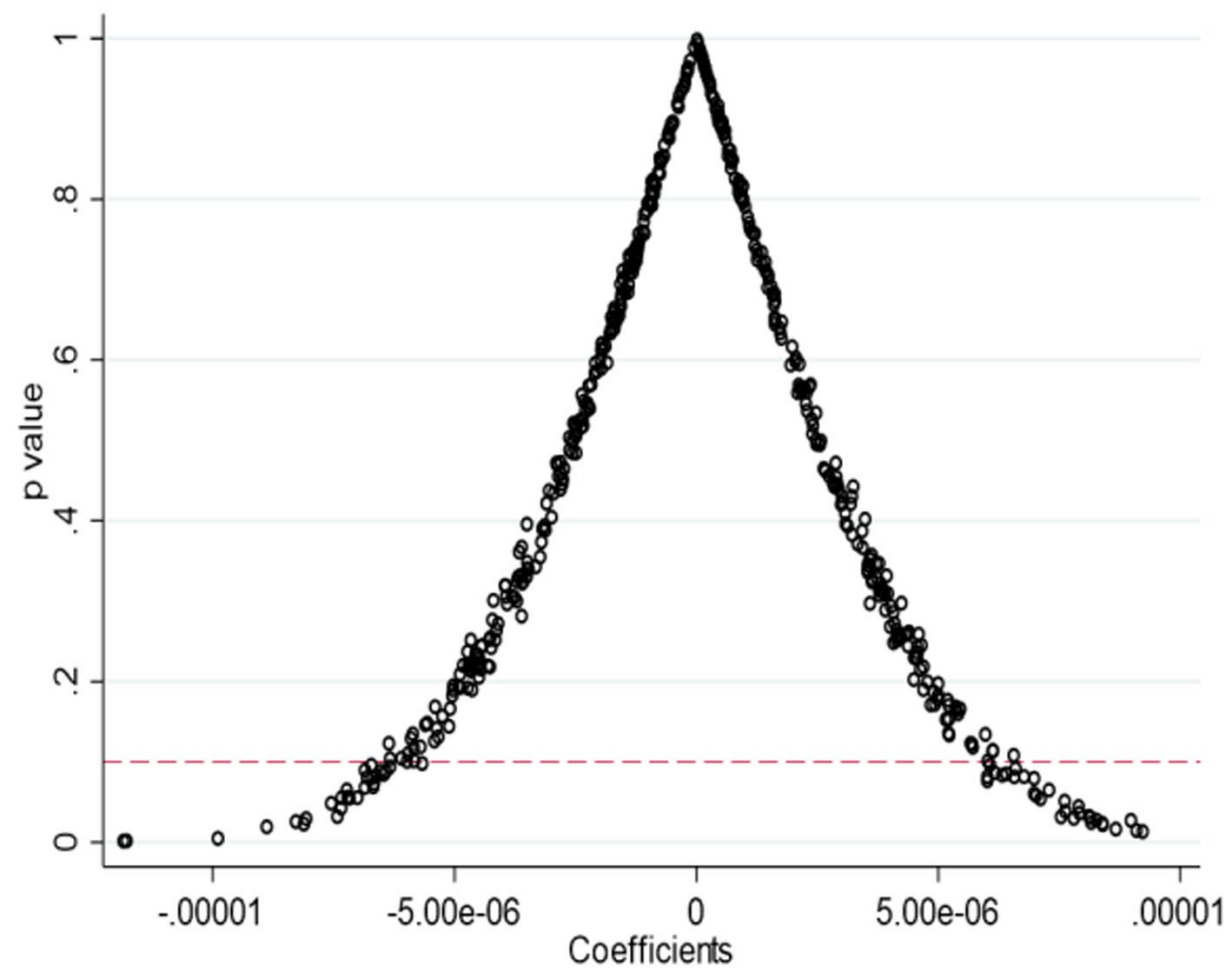
Placebo test.

## 6 Further analysis

### 6.1 Testing the shifting effect of enterprise SIC

#### 6.1.1 Estimating the elasticity of labor demand and labor supply

Theoretical analysis in the preceding sections suggests that the strategy enterprises adopt to alleviate the burden of SIC depends on the relative elasticity of labor supply and labor demand in the labor market. Therefore, this paper employs the estimation methods proposed by Li and French and Stafford to estimate labor supply elasticity and labor demand elasticity, respectively, using A-share listed companies’ data from 2011 to 2019 and the China Household Finance Survey (CHFS) data from 2011 to 2019 (34, 35). The method for estimating labor supply elasticity is as follows:

Step 1: Specify the following econometric model to estimate individuals’ potential wage levels:

$$\begin{array}{l} \mathrm{Ln}W_{i}=\varphi_{0}+\varphi_{1}\mathrm{Ed}u_{i}+\varphi_{2}\mathrm{Ag}e_{i}+\varphi_{3}\mathrm{Ag}e_{i}^{2}\\ +\varphi_{4}\text{Gende}r_{i}+\varphi_{5}\text{Huko}u_{i}+\varphi_{6}Z_{i}+\varepsilon_{i}. \end{array}$$ (3)

where individual i’s real wage level (*LnW_i_*) is determined by factors such as educational attainment (*Edu_i_*), age (*Age_i_*), gender (*Gender_i_*), and household registration type (*Hukou_i_*).^1^ In addition, this paper controls for provincial and year fixed effects as *Z_i_*. Based on Equation (3), this study utilizes data from CHFS 2011–2019 to estimate all parameters. Using these estimates, the potential wage levels of individual employees ( $\tilde{\mathrm{Ln}W_{i}}$ ) are then simulated across the entire samples.

Step 2: Use the Probit method to estimate the impact of wage changes on individual labor market participation and to calculate the wage elasticity of labor force participation. The model is as follows:

$$P({\text{Work}}_{i}=1)=\Phi (\gamma_{0}+\gamma_{1}\tilde{{\mathrm{LnW}}_{i}}+\gamma_{2}X_{i}+\epsilon_{i}$$ (4)

where *P*(*Work_i_* = 1) represents the probability of labor force participation for individual *i*, and Φ(⋅) denotes the probability distribution function. Parameters are estimated using the maximum likelihood estimation method. The variable *Work_i_* indicates whether an individual participates in the labor market, coded as a binary variable (1 for labor participation and 0 otherwise). *X_i_* captures other factors influencing an individual’s labor participation decision, including education level, age, age squared, gender, household registration type, marital status, political affiliation, health status, nature of the employing unit, household per capita income, social insurance participation, as well as province and year fixed effects.^2^ Through the estimation of this model, the marginal effect of wages on individual labor participation can be derived, which represents the wage elasticity of labor supply.

For the estimation of demand elasticity, the following model is specified:

$$\begin{array}{l} {\text{LnEmp}}_{\mathrm{it}}=\theta_{0}+\theta_{1}{\mathrm{LnW}}_{\mathrm{it}}+\theta_{2}{\mathrm{LnR}}_{\mathrm{it}}\\ +\theta_{2}{\text{LnTFP}}_{\mathrm{it}}+\theta_{2}{\text{LnOutput}}_{\mathrm{it}}+\theta_{2}{\text{Eage}}_{\mathrm{it}}+\pi_{i}+\rho_{t}+\tau_{\mathrm{it}} \end{array}$$ (5)

where enterprise i’s labor employment decisions (*LnEmp_it_*) are influenced by factors such as wage levels (*LnW_it_*), capital prices (*LnR_it_*), total factor productivity (*LnTFP_it_*), output (*LnOutput_it_*), and the age of the enterprise (*LnEage_it_*).^3^ In addition, enterprise fixed effects and year fixed effects are included to account for unobserved heterogeneity.

To enhance comparability, all continuous variables mentioned above were standardized, ensuring that all variables fall within the range of 0 to 1. Based on Equation (5), this study employs A-share listed company data from 2011 to 2019 to estimate the coefficients for employee wages, which represent the labor demand elasticity.

The calculations reveal that during the implementation period of the SIL, the labor demand elasticity (0.977) is significantly higher than the labor supply elasticity (0.013).^4^ This suggests that, during the sample period, it was easier for enterprises to alleviate the burden of SIC by reducing the workforce rather than cutting wages. The comparison of elasticity coefficients is consistent with the baseline regression, further supporting Hypothesis 1.

#### 6.1.2 The impact of SIC on employee compensation

Previous research indicates that during the implementation period of the SIL, the labor demand elasticity is greater than the supply elasticity, making it more difficult for enterprises to shift the burden of SIC through wage reductions. As a result, enterprises are forced to resort to layoffs to alleviate the burden of SIC. To verify this argument, this paper further examines the impact of SIC resulting from the SIL on employee compensation.

Regarding the definition of employee compensation, this paper uses the ratio of cash paid to employees and for employees to the number of employees, derived from the financial statements of listed companies. The cash paid to employees and for employees includes actual wages, bonuses, various allowances and subsidies, as well as the “five insurances and one fund” and welfare expenses paid on behalf of employees, providing a comprehensive reflection of employee compensation. Furthermore, to account for the effects of inflation, this paper adjusts employee compensation to the 2019 price level using the GDP deflator, ensuring the accuracy of the estimated results.

Before conducting the empirical analysis, this paper first calculates the changes in employee compensation growth rates for the period 2008–2016 using A-share data from Chinese listed companies. Figure 5 shows that the growth rate of employee compensation did not decline after 2011 but instead continued to increase. This initially suggests that, in response to rising SIC, enterprises do not resort to wage reductions to cope with this labor cost pressure.

**Figure 5:**
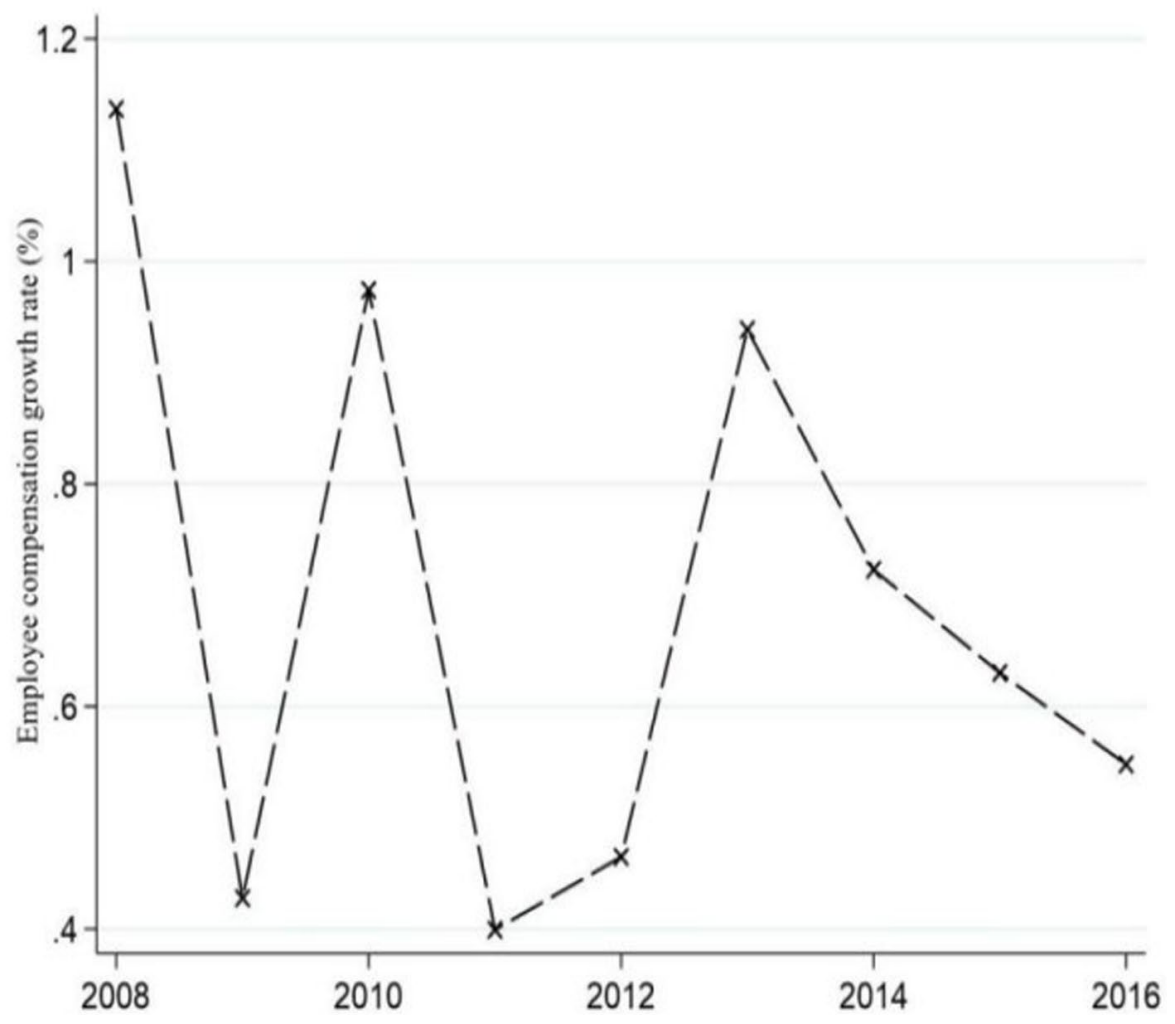
Change of employee compensation growth rate.

Furthermore, Table 7 presents the estimated results of the impact of increased SIC, caused by the SIL, on employee compensation levels. To ensure the robustness of the estimation results, this paper employs a stepwise regression approach, sequentially adding different control variables into the estimation equation. Column (1) presents the results of the effect of SIC on employee compensation without considering other factors. The result indicates that after the implementation of the SIL, in response to the increased burden of SIC, enterprises did not reduce employee compensation. Further, to account for the effects of province, industry, and firm-specific characteristics on employee compensation, and to mitigate omitted variable bias, the paper progressively includes province fixed effects, industry fixed effects, and relevant firm-specific variables into the estimation equation. The results, shown in Columns (2)–(4), indicate that the coefficient of Treat*Post remains significantly positive. Economically, using Column (4) as an example, the estimated result shows that the increase in SIC led to a 6.51% increase in employee compensation. This suggests that, during the sample period, due to the greater labor demand elasticity compared to labor supply elasticity, the difficulty in shifting the burden of SIC was high, and enterprises did not use wage reductions as a means to offset these costs. Instead, they were more likely to reduce labor employment levels to alleviate the burden of SIC. Hypothesis 1 is fully verified.

**Table 7:** Impact of SIC on employee compensation.

	Baseline	+ Controls	+Province FE	+Industry FE
	(1)	(2)	(3)	(4)
Treat*Post	0.0501^**^	0.0501^**^	0.0501^**^	0.0631^***^
	(0.0231)	(0.0231)	(0.0232)	(0.0230)
Control variables		Yes	Yes	Yes
Province FE			Yes	Yes
Industry FE				Yes
Enterprise FE	Yes	Yes	Yes	Yes
Year FE	Yes	Yes	Yes	Yes
*N*	11,868	11,868	11,868	11,868
Adjusted-*R*^2^	0.9997	0.9997	0.9997	0.9997
F-statistic	4.7164	4.7045	4.6770	4.5265

*, **, and *** represent significance at the 10, 5, and 1% levels, respectively. The enterprise-level cluster robust standard errors are in parentheses.

The difficulty of shifting the SIC burden onto employees during the sample period can be explained from two perspectives: efficiency wages and labor protection. From the perspective of efficiency wage theory, when facing an increase in SIC, if enterprises attempt to alleviate the burden by reducing wages, two unfavorable consequences may arise. First, a decrease in employees’ wages will reduce their motivation to work (36), which in turn negatively affects the enterprise’s productivity. Second, a wage reduction may lead to employees “voting with their feet” and quitting the enterprise (37), which would seriously disrupt the stability of the enterprise’s production order and lower output levels. In other words, while wage cuts might appear to help enterprises reduce labor costs and alleviate the SIC burden, this action could simultaneously reduce the enterprise’s output, resulting in a “penny wise, pound foolish” situation. Therefore, from the efficiency wage theory perspective, enterprises are not necessarily inclined to reduce wages in response to higher SIC.

From the perspective of labor protection, the ability to shift SIC depends on labor-capital bargaining power. Although employers generally hold greater bargaining power than employees in labor markets, the bargaining power of workers has significantly increased with the improvement of labor protection systems, such as the implementation of the revised Labor Contract Law in 2008 and the strengthening of trade unions’ role in defending workers’ rights (38). If enterprises try to reduce wages to mitigate the increased SIC, it will inevitably harm the rights of employees. In such cases, the Labor Contract Law and the role of trade unions can effectively restrain the enterprises’ wage-cutting practices. Therefore, enterprises are also unlikely to use wage reductions to offset the increased SIC burden.

### 6.2 Mechanism test

#### 6.2.1 Liquidity constraint

Theoretical analysis suggests that after the implementation of the SIL, the increased SIC will crowd out the available funds for enterprises, increase the difficulty of signing credit contracts with banks, and intensify liquidity constraints, thereby prompting enterprises to reduce labor employment. Therefore, this study further examines whether the increased SIC resulting from the SIL exacerbates the liquidity constraints of enterprises. In terms of liquidity constraint indicators, this study uses two measures: the net cash flow from operating activities (Constraint 1, expressed in natural logarithms) and the ratio of net cash flow from operating activities to enterprise liabilities (Constraint 2).

The estimation results are shown in Table 8. After gradually adding relevant control variables, the increased in SIC significantly reduces both the net cash flow from operating activities and the ratio of this indicator to enterprise liabilities. The increased SIC exacerbates the liquidity constraints of enterprises, thereby prompting them to reduce labor employment. Hypothesis 2 is validated.

**Table 8:** Liquidity constraint mechanism.

	Constraint 1	Constraint 1	Constraint2	Constraint 2
	(1)	(2)	(3)	(4)
Treat*Post	−0.6237^***^	−0.6412^***^	−0.0590^***^	−0.0357^***^
	(0.1996)	(0.2044)	(0.0136)	(0.0118)
Control variables		Yes		Yes
Province FE		Yes		Yes
Industry FE		Yes		Yes
Enterprise FE	Yes	Yes	Yes	Yes
Year FE	Yes	Yes	Yes	Yes
*N*	13,886	13,886	13,904	13,904
Adjusted-*R^2^*	0.9286	0.9283	0.4555	0.5124
F-statistic	9.7578	7.0914	18.7241	59.8080

*, **, and *** represent significance at the 10, 5, and 1% levels, respectively. The enterprise-level cluster robust standard errors are in parentheses.

#### 6.2.2 Production scale

The increase in SIC raises labor costs for enterprises, which in turn reduces their profit margins, undermines production incentives, and potentially leads to a contraction in production scale. One prominent manifestation of this is the reduction in labor employment. Therefore, this study further examines whether the rise in SIC encourages enterprises to scale back their production. In terms of the production scale indicators, this paper adopts two approaches: First, following Subedi and Farazmand, the sum of value-added labor compensation, net production taxes, operating profits, and depreciation of fixed assets to represent production scale is used (Scale 1) (39); Second, enterprise sales revenue is used as an indicator (Scale 2).

As can be observed in Table 9, after progressively adding relevant control variables, the increased SIC resulting from the implementation of the SIL significantly reduces the production scale of enterprises. This result is likely to manifest as a reduction in labor employment. Hypothesis 3 is validated.

**Table 9:** Production scale mechanism.

	Scale 1	Scale 1	Scale 2	Scale 2
	(1)	(2)	(3)	(4)
Treat*Post	−0.5919^***^	−0.5999^***^	−5.0283^***^	−5.5108^***^
	(0.1358)	(0.1284)	(0.7734)	(0.7569)
Control variables		Yes		Yes
Province FE		Yes		Yes
Industry FE		Yes		Yes
Enterprise FE	Yes	Yes	Yes	Yes
Year FE	Yes	Yes	Yes	Yes
*N*	12,718	12,718	12,718	12,718
Adjusted-*R*^2^	0.9026	0.9162	0.8730	0.8850
F-statistic	19.0012	25.6300	42.2724	13.4394

Both production scale indicators are in the form of natural logarithms. *, **, and *** represent significance at the 10, 5, and 1% levels, respectively. The enterprise-level cluster robust standard errors are in parentheses.

#### 6.2.3 Capital substitution

The increase in SIC raises the cost of labor, making capital relatively cheaper. As a result, enterprises may be inclined to increase capital input to substitute for the more expensive labor input, leading to a reduction in labor employment. Therefore, this study further examines whether the rise in SIC resulting from the SIL prompts enterprises to engage in capital substitution behavior. To measure capital substitution, this paper uses the natural logarithm of the capital-labor ratio (net fixed assets per employee) as the indicator.

As shown in Table 10, after progressively adding relevant control variables, the increased SIC significantly raises the capital-labor ratio of enterprises. Notably, the coefficient in Column (4) (0.0987, *p* < 0.05) is smaller than those in Columns (1)–(3) (approximately 0.129, *p* < 0.01). If the increase in the capital-labor ratio were purely mechanical—driven solely by the reduction in employment (the denominator)—the coefficient would be expected to remain constant across specifications. The observed attenuation pattern is therefore more consistent with a genuine behavioral response that is partially absorbed by firm characteristics.

**Table 10:** Capital substitution mechanism.

	Progressively adding control variables			
	(1)	(2)	(3)	(4)
Treat*Post	0.1293^***^	0.1293^***^	0.1289^***^	0.0987^**^
	(0.0430)	(0.0431)	(0.0432)	(0.0414)
Control variables				Yes
Province FE			Yes	Yes
Industry FE		Yes	Yes	Yes
Enterprise FE	Yes	Yes	Yes	Yes
Year FE	Yes	Yes	Yes	Yes
*N*	13,903	13,903	13,903	13,903
Adjusted-*R*^2^	0.7893	0.7881	0.7877	0.8108
F-statistic	9.0436	9.0000	8.9189	23.3304

*, **, and *** represent significance at the 10, 5, and 1% levels, respectively. The enterprise-level cluster robust standard errors are in parentheses.

We acknowledge that the capital-labor ratio may partly increase mechanically due to the reduction in employment, as established in the baseline results. Therefore, the results in Table 10 should be interpreted as suggestive evidence consistent with capital deepening, rather than definitive proof of active capital substitution. In this sense, these results are consistent with Hypothesis 4.

### 6.3 Heterogeneity analysis

#### 6.3.1 Financing constraints

The implementation of the SIL and the resulting increase in SIC raises labor costs for enterprises, potentially exacerbating their financing constraints. In such cases, enterprises may resort to layoffs as a coping mechanism. Therefore, compared to enterprises with more relaxed financing constraints, those facing severe financing constraints are likely to implement more significant layoffs. This study further examines how SIC affect labor employment in enterprises with different degrees of financing constraints.

In the setting of the financing constraint indicator, this study follows the approach of Almeida et al. and Hadlock and Pierce by using the SA index to measure the degree of financing constraints faced by enterprises (40, 41)^,^ where a higher value indicates more severe financing constraints.^5^ Specifically, enterprises with an *SA* index above the median are categorized as facing more severe financing constraints, while those with an SA index at or below the median are categorized as facing more relaxed financing constraints.

The estimation results are presented in Columns (1) and (2) of Table 11. After the implementation of the SIL, the increased SIC significantly reduced the labor employment scale of enterprises facing severe financing constraints, but did not affect the labor employment decisions of enterprises with more relaxed financing constraints.

**Table 11:** Heterogeneity analysis.

	Financing constraints		Scale of labor employment	
	Low	High	Small	Large
	(1)	(2)	(3)	(4)
Treat*Post	0.0243	−0.1988^***^	−0.0599	−0.0750^**^
	(0.0615)	(0.0604)	(0.0579)	(0.0374)
Control variables	Yes	Yes	Yes	Yes
Province FE	Yes	Yes	Yes	Yes
Industry FE	Yes	Yes	Yes	Yes
Enterprise FE	Yes	Yes	Yes	Yes
Year FE	Yes	Yes	Yes	Yes
*N*	6,888	6,890	6,888	6,883
Adjusted-R^2^	0.8201	0.8897	0.7582	0.9130
F-statistic	46.4426	32.4294	27.7600	48.7534

*, **, and *** represent significance at the 10, 5, and 1% levels, respectively. The enterprise-level cluster robust standard errors are in parentheses.

#### 6.3.2 Scale of labor employment

When enterprises consider reducing their labor employment scale, the larger the labor employment scale, the greater the room for layoffs. Therefore, compared to enterprises with smaller labor employment scales, after SIL, the increased SIC will significantly reduce the labor employment numbers of enterprises with larger labor employment scales. To examine this, this study further investigates the impact of the increase in SIC on the employment numbers of enterprises with different labor employment scales.

In the categorization of labor employment scale groups, the samples are divided into two groups based on the median of labor employment numbers: enterprises with a larger labor employment scale (above the median) and those with a smaller labor employment scale (at or below the median). Empirical tests are then conducted for each group.

In Columns (3) and (4) of Table 11, the findings indicate that after the SIL, the increased SIC significantly reduced the labor employment numbers of enterprises with larger labor employment scales, but did not affect the labor employment decisions of enterprises with smaller labor employment scales.

Beyond the two dimensions examined above, climate exposure and industry affiliation constitute additional relevant sources of heterogeneity. It should be noted that the industry dimension is partially embedded in the research design: the treatment and control groups are defined by labor intensity, so that the treatment group consists predominantly of labor-intensive manufacturing firms, whereas the control group contains firms with low labor intensity, including low-labor services; the baseline contrast thus already captures a substantial part of the industry heterogeneity. With respect to climate exposure, firms located in heavily polluted regions or areas frequently affected by extreme weather face more intense climate-related health pressures, and the employment impact of rising SIC is plausibly more pronounced for these firms. However, firm-level measures of climate exposure are not available in the dataset of Chinese listed companies used in this study; constructing such measures would require matching external meteorological and environmental data at the city level, which is beyond the scope of the current paper. We acknowledge this as a limitation and leave the climate-exposure heterogeneity of the relationship between SIC and labor employment to future research.

## 7 Conclusion

In the context of increasing economic downward pressure, achieving the goal of stabilizing employment has become an urgent issue that China’s government needs to address. This paper takes the implementation of China’s SIL in 2011 as a quasi-natural experiment and uses A-share data from Chinese listed companies from 2007 to 2019. It empirically analyzes the impact of SIC on enterprise labor employment decisions and its mechanisms using a DID model. The study finds the increase in SIC levels significantly leads to a reduction in enterprise labor employment scales. The analysis of the shifting effect shows that, compared to reducing wages, enterprises are more likely to alleviate the burden of SIC through layoffs. Furthermore, the rise in SIC levels exacerbates enterprises’ liquidity constraints, prompting them to reduce production scale or accelerate capital substitution for labor, which also negatively affects labor employment numbers.

Our empirical results provide robust support for the proposed hypotheses. Consistent with Hypothesis 1a, we find that the labor demand elasticity significantly exceeds labor supply elasticity during the sample period, leading firms to prefer layoffs over wage reductions to cope with rising SIC. Furthermore, the confirmation of Hypotheses 2 through 4 substantiates the multifaceted pathways—liquidity constraints, production scale contraction, and capital substitution—through which SIC burdens dampen employment. These results are in line with the studies by Acemoglu and Restrepo (13), who document capital-labor substitution in response to rising labor costs, and Benzarti and Harju (21), who show that payroll tax increases lead to significant employment reductions. At the same time, our findings extend the work of Kugler and Kugler (17) by demonstrating that, in the Chinese context, the layoff channel dominates the wage adjustment channel, a distinction that is crucial for understanding the heterogeneous labor market responses to social security policies.

Although the social insurance fee reduction policy was implemented in 2019, it remains to be verified whether the extent of the reduction is sufficient to effectively alleviate the burden on enterprises. Therefore, there may be a need to further moderate the reduction in SIC rates in order to ease the pressure on enterprises and promote employment stability, thereby advancing the goal of stabilizing employment. On the other hand, the SIC policy should be more precise and targeted, particularly by considering the financing constraints and labor employment scales faced by different enterprises and formulating differentiated policies to ensure that the policy effects are maximized. In addition, the government should provide more support to enterprises, to help them alleviate the pressure caused by SIC. This can be achieved through tax incentives, fiscal subsidies, and other measures to ensure employment stability.

This study has certain limitations in sample representativeness, policy shock identification and employment outcome measurement. The sample is confined to China’s A-share listed companies, and this paper focuses on the aggregate scale of labor employment without further exploring employment structural changes or directly incorporating climate risk indicators to test their moderating effects. Future research can expand the sample to cover more small and medium-sized and non-listed enterprises, decompose the heterogeneous effects of different social insurance policy tools, and examine the joint impact of climate uncertainty and social security costs on labor market outcomes. Future research could also employ more direct measures of capital investment (e.g., new fixed asset purchases, R&D expenditure) to better isolate the capital substitution channel from the mechanical composition effect, so as to provide more comprehensive empirical support for policy formulation.

## Data Availability

The raw data supporting the conclusions of this article will be made available by the authors, without undue reservation.

## Footnotes

1 The wage level of individual employees is represented by the natural logarithm of annual per capita household income. Educational attainment is categorized into seven levels: illiterate = 1, primary school = 2, junior high school = 3, senior high school = 4, technical secondary school = 5, junior college/vocational education = 6, and undergraduate and above = 7. Gender is represented by a binary variable, with male = 1 and female = 0. For household registration type, rural registration is coded as 0 and urban registration as 1.

2 Labor participation is a binary variable: employment = 1 and unemployment = 0. Marital status: 1 for married = 1 and unmarried = 0. Political affiliation: party members = 1 and non-members = 0. Health status is categorized into five levels: very poor = 1, poor = 2, average = 3, good = 4, and very good = 5. The nature of the employing unit is divided into government/public sector organizations (valued as 1) and non-government/private entities (valued as 0). Household per capita income is expressed in natural logarithms. Social insurance participation is coded as 1 if the individual is enrolled in pension or health insurance and 0 otherwise.

3 Labor employment is measured as the natural logarithm of the number of employees. Wage levels are represented as the natural logarithm of the ratio of total cash payments made to employees to the number of employees. Capital price is proxied by the natural logarithm of the firm’s fixed assets. TFP is calculated using the Solow residual method and expressed in natural logarithms. Output is defined as the sum of total cash payments to employees, net production taxes, operating profits, and fixed asset depreciation, and it is also expressed in natural logarithms.

4 The elasticity of labor supply measured by the wage income of individuals is 0.575, which is also less than the elasticity of labor demand.

5 ^2^*SA* = –0.737 * *Size* + 0.043 * *Size* – 0.040 * *Eage*, where, *Size* is the natural logarithm of the enterprise size, and *Eage* is the years that the enterprise was established.
